# Prioritizing the Potential Applications of Mobile-Health in the Iranian Health System

**DOI:** 10.34172/jrhs.2020.08

**Published:** 2020-03-01

**Authors:** Mina Noee, Ali Akbari Sari, Alireza Olyaeemanesh, Mohammadreza Mobinizadeh

**Affiliations:** ^1^Department of Health Economics and Management, School of Public Health, Tehran University of Medical Sciences, Tehran, Iran; ^2^National Institute of Health Research and Health Equity Research Centre, Tehran University of Medical Sciences, Tehran, Iran; ^3^National Institute for Health Research, Tehran University of Medical Sciences, Tehran, Iran

**Keywords:** Mobile-health, M-health, Priority setting

## Abstract

**Background:** Access and the use of information and communication technology, especially mobile phones, have expanded significantly in recent years; therefore, we aimed to rank the potential applications of mobile apps in the Iranian health system.

**Study Design:** A multi-attribute decision making design.

**Methods:** First, the main applications of apps and also the related attributes for prioritization were extracted from a systematic and comparative review of studies. Then, the weight of these attributes was extracted using the Shannon Entropy method. The values of attributes for each application were questioned by the 11 experts. By having the decision matrix and the weight of attributes, the applications were separately weighted and ranked using four MADM techniques. Finally, using the Copeland technique, the results of different techniques were combined, and a final ranking was achieved.

**Results:** Based on the results extracted from the studies and the opinions of experts, 8 main applications, and, 14 attributes were determined and entered into the modeling phase. The most significant weight obtained was related to "the feasibility of monitoring activities" (weight=0.220), and the least was related to "the feasibility of access to apps in any location" (weight=0.017).

**Conclusion:** The apps related to the physicians' access to patients’ health information had the highest priority, and the apps related to the selection of proper health behavior patterns had the least priority.

## Introduction

Information technology, as a powerful tool, is considered as the most critical factor for productivity improvements in the organizations. Therefore, given the role and importance of the health care industry and its effects on society, information technology has been considered as a tool for improving the level of people’s health. The term e-health is a broad term used since 2000 and includes different aspects of using technologies and E-commerce infrastructures. According to the WHO's definition, e-health emphasizes the cost-saving and safe use of information and communication technologies in support of health care including health care services, supervision, educational, knowledge and related research services^1^. Moreover, e-health communication was an important tool for promoting of healthy lifestyle ^2^.

The concept of mobile health was considered as a sub-category of electronic health, emerged in 2003 due to the easy portability of mobile phones while connecting to the Internet ^1^. Mobile applications are apps designed for electronic devices such as smartphones and tablets. The empowering of an individual to receive available treatments, maintaining people's participation in control and identification of symptoms, treatment, and provision of personal feedback and motivational support can be noted as the advantages of using this type of care ^3^.

The application of mobile health services has various dimensions. Some have been related to fitness and nutrition purposes. Some related to the executive aspects of the health care industry and some are used to change a patient's behavior or treat illnesses by providing tools to assess patients ^4^. Hence, mobile health applications have many benefits such as, facilitating access to health care, reducing social costs related to the illness and diversifying health services ^5^.

Three factors of physician, patient, and disease have essential roles in the treatment process of diseases. Out of these three factors, if two factors of physician and patient get united, they can get over the third factor, the disease. For uniting the two factors of physician and patient, it is necessary to establish a connection and interaction between them, for which mobile technologies can be used ^1^.

According to the available statistics in 2012, 84% of smartphone users had downloaded at least one health-related app ^6^. Moreover, the acceptance of technologies in health systems within the last decades has experienced some changes and public and policy makers have an ambiguous idea about the use of new health technology. Hence the designing a framework for priority setting of new technology which was useful for society especially in health sector will be important and essential ^7^.

Based on the researcher’s initial searches for identification of research gaps in terms of m-health, so far, in the domestic studies, the potential applications of mobile apps in the Iranian health system have been rarely studied. Therefore, this research was performed for designing a robust framework for priority setting of potential mobile apps for better health policy making in this regard.

## Methods

This comparative study was conducted by operational research modeling through multi-attribute decision-making. The main applications of apps and also the prioritizing attributes are extracted through a systematic and comparative review of studies. For this purpose, the critical related databases such as Scopus and PubMed were searched, and in the next step, the articles were excluded based on the studied title and unnecessary items.

The articles obtained from this step were studied based on the specified inclusion criteria and entered the final phase of the study. The data obtained from the included articles was entered into a comparative table, and after the declaration of experts’ opinions, the common attributes were suggested for Iran. After initial search of studies and matching the included studies, the applications and attributes identified were presented to the health information technology experts through the questionnaires of relevancy and necessity in the first step and the importance questionnaire in the second step, and finally, the attributes and applications gained the most importance entered the modeling phase (MADM). Hence the following steps were done for performing this research.

### Specifying the main application and attributes

In this step, after conducting a systematic and comparative review study, the main important application and attributes in the prioritizing of mobile applications in the field of health were identified. The systematic review was performed for identification of the main applications of mobile apps related to healthcare and also proper attributes for designing priority setting framework. For this purpose, the main databases such as PubMed, Scopus, and Google Scholar were searched with the proper search strategy. After definition of the inclusion criteria for reviewing of retrieved papers, the self-made data extraction form was used to extract data from retrieved researches.

### Extraction of final applications and attributes

The statistical population of the study consisted of health system experts who had full knowledge of the mobile apps and technologies related to the mass communication. The sample size of the study was selected based on available experts.

In this step, the main important application and attributes in the prioritizing of the health apps were presented to the health information technology experts. Then, after extraction of opinions of 11 experts in the field of health information technology (all experts were related to Tehran University of Medical Sciences which either academic members or health professionals who worked in hospitals or health centers), two criteria of relevancy and necessity were calculated for each attribute. The attributes were entered into the final phase of importance determination that had a cut-off point of 75% for relevancy and 75% for necessity. Moreover, in the next phase of importance determination, a cut-off point of 75% was considered for determination of main apps and attributes for inclusion on final priority setting framework.

After determining the final attributes impacting the selection of health app applications, their weight was calculated. The Shannon entropy method was used for this purpose. In this method, the attributes gain more weight that the experts’ opinions or evidence about them have more diversity and disparity, which indicates that more attention should be paid to that attribute.

### Quantification of qualitative attributes

Since the final attributes for each application are assessed qualitatively, their quantitative equivalents were designed in this section. For each of the final attributes, self-made scales were designed and used by reviewing the resources.

### Prioritizing the final applications

The weight of attributes in this model was obtained through Shannon entropy weighting technique, and then, using the obtained weights, 4 main models of SAW: Simple Additive Weighting (The simplest method of MADM based on weighted average), TOPSIS: The Technique for Order of Preference by Similarity to Ideal Solution (In this method, the ranking of each alternative rely on its closeness to the positive ideal solution and the negative ideal solution), VIKOR: VlseKriterijumska Optimizacija I Kompromisno Resenje (In this method, the ranking of each alternative rely on special approach for measuring of ‘‘closeness” to the ‘‘ideal” solution) and ELECTRE 1: ELimination Et Choix Traduisant la REalité (In this method, the preference of an alternative over the other alternatives was calculated by special approach for pairwise comparison of alternatives) were used for prioritizing the applications, and finally, the Copeland combined method was used for the conclusion and extraction of a general prioritization. In this method, a paired comparison matrix is created between the options for decision making. Based on the different multi-attributes decision making techniques, if the priority number of an option on another option is more than its defeat number, the letter M is placed in the paired comparison matrix, and if there is no majority of the votes are equal, the letter X is placed in the paired comparison matrix. Then, the number of dominances of options (total elements of each row) and the number of defeats of options (total elements of each column) are calculated. In the end, the options are ranked in an ascending order based on the difference between the number of defeats and the number of dominances.

## Results

### Systematic and comparative review

In the first search, 84 papers were retrieved (26 papers from Scopus, 57 ones from PubMed and one from Google Scholar). Finally, after excluding of duplication and compliance with inclusion criteria, 9 articles remained for final analysis (Table 1). All extracted data were analyzed through thematic synthesized. The main themes for mobile apps were apps related to behavioral changes, apps related to data gathering, apps related to the health system management and apps related to health service delivery. And the main themes for attributes were information system quality, health service quality and other general attributes for priority setting.

**Table 1:** List of final studies included in the research

**No.**	**Title**	**First Author**	**Year**
1	A Framework for Selecting Digital Health Technology ^8^	Andrey Ostrovsky,	2013
2	Evaluating and selecting mobile health apps: strategies for healthcare providers and healthcare organizations ^9^	Edwin D Boudreaux	2014
3	What is the economic evidence for mHealth? A systematic review of economic evaluations of mHealth solutions ^10^	Sarah J. Iribarren	2017
4	The health systems' priority setting criteria for selecting health technologies: A systematic review of the current evidence ^11^	Mobinizadeh	2016
7	Evaluating mobile medical application ^12^	Conor Hanrahan	2014
8	Mobile Health Services: Past, Present, Future ^1^	Ahmadizad, Varmaghani	2017
9	Consumer Mobile Health Apps: Current State, Barriers, and Future Directions ^13^	Kao, Cheng-Kai	2018
10	Cognitive factors of using health apps: systematic analysis of relationships among health consciousness, health information orientation, eHealth literacy, and health app use efficacy ^6^	Cho, Jaehee	2014
11	The Role of Mobile Applications in Delivery of Mental Health Services: A Review Study ^3^	Borjalilu Somaye	2016

### Extraction of Final Applications

Following extraction of main applications from retrieved papers, the extracted applications were questioned from 11 experts (They worked in Tehran University of Medical Sciences and had 35 to 60 yr old) in 3 phases (Relevancy, Necessity and Importance). Finally, the following 8 main applications were included into the MADM phase; “Apps related to the improvement of social awareness and behavior on health issues”, “Apps related to the increased knowledge of patients about disease”, “Apps related to the selection of appropriate health behavioral patterns”, “Apps related to the recording and tracking of vital signs”, “Apps related to the access of physicians to health information of patients”, “Apps related to the improvement of the service quality”, “Apps related to the provision of health care for remote areas”, “Apps related to the support of electronic decision-making in health-related issues”.

### Extraction of final attributes

Following extraction of related attributes from retrieved papers, the extracted attributes were questioned from 11 experts in 3 phases (Relevancy, Necessity and Importance). Finally, the following 14 attributes were included into the MADM phase; “The feasibility of monitoring activities”, “The feasibility of learning and user interface”, “Existence of proper system support”, “System response time”, “The feasibility of access to apps in any location”, “The feasibility of making fast and efficient communication”, “Level of system privacy and security protection”, “Existence of the user guide”, “Level of service quality”, “Ability to adjust; upgrade and update”, Safety, Efficacy / Effectiveness, “Level and quality of clinical evidence regarding the application” and “Economic aspect regarding the application”.

### Determining the weights of attributes

The results of the weights obtained through Shannon entropy method were such that “the feasibility of monitoring activities” had the most significant weight (weight=0.220) and “the feasibility of access to apps in any location” had the least weight (weight=0.017) (Table 2).

**Table 2:** The weights of attributes

**Attribute**	**Shannon entropy weight**
The feasibility of monitoring activities	0.220
The feasibility of learning and user interface of	0.077
Existence of proper system support	0.075
System response time	0.065
The feasibility of access to apps in any location	0.017
The feasibility of making fast and efficient communication	0.121
Level of system privacy and security protection	0.090
Existence of the user guide	0.032
Level of service quality	0.041
Ability to adjust; upgrade and update	0.043
Safety	0.022
Efficacy/Effectiveness	0.034
Level and quality of clinical evidence regarding the application	0.034
Economic aspect regarding the application	0.131

### Forming the decision matrix

Since there are 8 applications and 14 attributes in this study, thus the decision matrix is an 8×14 matrix. In order to form the decision matrix, codes, and scales were designed and extracted for each application of m-health (Table 3).

**Table 3:** The extracted codes for each attribute

**Attribute /Levels**	**Scale**
**The feasibility of monitoring activities**	
Monitoring activities of health service provider centers increases the satisfaction and well-being of users	5
Monitoring activities of health service provider centers lead to the better and more cohesive data store	4
Monitoring activities of health service provider centers improve the quality of services to users through storing and on the base of users' specific needs	3
Low level of monitoring over the activities	2
Lack of monitoring of activities	1
**The feasibility of learning and user interface**	
The user has a pleasant experience and feel when using the app and to have a positive impact on the user.	5
The user interface has a good design in a way to encourage the user to download and use the app more.	4
Applications with a more familiar platform for the use of users to provide better user experience, such as HTML, CSS, and 5JS.	3
Having the ability to run on different devices and the possibility of personalizing the app.	2
Can only be installed on some devices	1
**Existence of proper system support**	
Supported by the Ministry of Health	5
Supported by some private hospitals or private IT centers	4
Supporting only Android and IOS	3
Supported by many preconditions	2
No support	1
**System response time**	
Having a fast system response during the use of users to increase the satisfaction of mobile users while ensuring quality and accuracy in performance	5
Reduce the time spent on providing services and not waiting for the user	4
Relatively fast system response time with medium quality	3
Slow response to the user request	2
Responding to the user request without considering the response time	1
**The feasibility of access to apps in any location**	
Increasing the rate of easy access of more people in the society to services in any location resulted in the costs’ control, correct management of health measures, and health promotion.	5
Increasing the rate of easy access of more people in the society to services in any location resulted in the decrease of disease severity in the long term.	4
Sending the user requested data at any time and place	3
Difficult access at any location	2
Tough access at any location	1
**The feasibility of making fast and efficient communication**	
Supporting high-speed and efficient communications to obtain information simultaneously to provide the best treatment method	5
Supporting fast and efficient communications to increase and combine the clinical knowledge of service provider staff	4
Supporting somewhat fast and efficient communications to accelerate the processes of diagnosis	3
Supporting communications for information on disease records	2
Lack of fast and efficient communications in various aspect	1
**Level of system privacy and security protection**	
Using internal memory to store sensitive data	5
Encrypting data in the external storage	4
Using the Internet for IPC (Inter-Process Communication) that are a set of methods for exchanging information between several macros and micro-processes (or threats).	3
Using outdated electronic data protection systems	2
No specific system	1
**Existence of the user guide**	
A user guide with adaptability and flexibility to facilitate using the application services and increase the knowledge on using with a high performance	5
A manual for attracting users and retaining them with a moderate performance	4
A simple and clear manual without any complexity and with low performance	3
A simple, general manual	2
Lack of manual	1
**Level of service quality**	
Helps and facilitates health services’ delivery	5
Improves the performance health services’ delivery	4
Facilitates the possibility of exchanging information between healthcare institutions.	3
Reduces medical errors and mistakes.	2
Provide users with better treatment practices.	1
**Ability to adjust; upgrade and update**	
Providing daily updates	5
Providing personalized settings	4
Upgrading the capabilities	3
Lack of updating	2
No personalized settings	1
**Safety**	
No side effect	5
Mild and slide side effect with low incidence	4
Mild and slide side effect with a moderate incidence	3
Mild and slide side effect with a high incidence	2
The moderate side effect with low incidence	1
**Efficacy / Effectiveness**	
Improves their quality of life.	5
Improves care outcomes.	4
Increases self-care in individuals	3
Improves motivation to continue the treatment	2
Increases patients’ satisfaction	1
**Level and quality of clinical evidence regarding the application**	
A systematic review of RCTs (Randomized Controlled Trials)	5
Randomized Controlled Trials	4
A systematic review of observational studies	3
Clinical observations	2
Lack of adequacy of clinical evidence	1
**Economic aspect regarding the application**	
Helping to make a profit, reducing human resources, reducing treatment costs, reducing the time of hospitalization in hospitals and treatment centers, improving the patients' quality of life	5
Helping to increase the access of society to health services	4
Helping to create an affordable system accessible to everyone	3
Helping to reduce treatment costs	2
Helping to reduce the severity of the disease	1

The elements of this matrix, which are, in fact, the values of the attributes for each application, were extracted from the evidence upon the collective agreement of the experts on the results, and finally, the decision matrix was formed as Table 4. Each element in this matrix was calculated based on the average participating experts’ opinions on that element.

**Table 4:** Decision matrix

**Alternatives**	The feasibility of monitoring activities	The feasibility of learning and user interface of	Existence of proper system support	System response time	The feasibility of access to apps in any location	The feasibility of making fast and efficient communication	Level of system privacy and security protection	Existence of the user guide	Level of service quality	Ability to adjust; upgrade and update	Safety	Efficacy / Effectiveness	Level and quality of clinical evidence regarding the application	Economic aspect regarding the application
Apps related to the improvement of society awareness and behavior on health issues	3.6	3.9	3.7	3.9	4.2	3.5	3.8	3.8	4.4	4.1	3.7	3.6	3.4	3.7
Apps related to the increased knowledge of patients about disease	3.2	4.3	3.6	4.2	4.0	3.7	3.7	3.9	4.2	3.9	3.8	3.7	3.4	3.6
Apps related to the selection of appropriate health behavioral patterns	3.7	3.9	4.1	4.0	4.3	3.9	3.7	4.2	4.1	3.9	3.9	3.9	3.3	3.2
Apps related to the recording and tracking of vital signs	4.5	3.9	3.8	4.5	4.5	3.6	4.3	4.2	4.0	4.2	4.1	3.9	3.5	4.3
Apps related to the access of physicians to health information of patients	4.7	4.3	4.2	4.8	4.3	4.5	4.5	3.9	4.6	4.1	3.8	4.0	3.5	4.1
Apps related to the improvement of the service quality	3.6	3.4	3.8	4.2	4.1	4.2	3.9	4.1	4.4	3.6	3.9	3.9	3.7	3.7
Apps related to the provision of health cares for remote areas	4.1	3.7	4.0	4.5	4.2	4.4	4.3	4.4	4.7	4.2	4.1	4.1	3.8	3.9
Apps related to the support of electronic decision-making in health related issues	4.1	3.9	4.5	4.5	4.2	4.2	4.4	4.1	4.3	4.3	4.1	4.2	3.7	4.3

### Prioritizing the final Applications

In this section, using the selected four techniques, namely SAW, TOPSIS, VIKOR, and ELECTRE 1, the applications of apps in the field of health were separately prioritized. These rankings are given in Table 5.

**Table 5:** Ranking the related applications obtained from SAW, TOPSIS, VIKOR, ELECTRE 1 and Copeland

**Health Apps**	**SAW**	**TOPSIS**	**VIKOR**	**ELECTRE 1**	**Copeland**
Apps related to the improvement of social awareness and behavior on health issues	7	7	7	5	7
Apps related to the increased knowledge of patients about disease	8	8	8	6	8
Apps related to the selection of appropriate health behavioral patterns	6	6	6	5	6
Apps related to the recording and tracking of vital signs	3	2	4	2	2
Apps related to the access of physicians to health information of patients	1	1	1	1	1
Apps related to the improvement of the service quality	5	5	5	4	5
Apps related to the provision of health care for remote areas	4	4	3	3	4
Apps related to the support of electronic decision-making in health-related issues	2	3	2	2	3

### Merging the results using the Copeland method

As a result of considering the opinions of experts and combining the results of different methods, the most critical priorities for developing the mobile apps in the Iranian health field are apps related to the access of physicians to health information of patients, apps related to the recording and tracking of vital signs, apps related to the support of electronic decision-making in health-related issues, apps related to the provision of health cares for remote areas, apps related to the improvement of the service quality, apps related to the selection of appropriate health behavioral patterns, apps related to the improvement of society awareness and behavior on health issues, and apps related to the increased knowledge of patients about disease (Table 5).

## Discussion

At a time when mobile and mobile apps are closer to the public than any other media and device, it is necessary to identify the potential applications of mobile apps in the Iranian health system. The main purpose of the research was to investigate the potential applications of mobile apps in Iranian health system. Eight main applications and 14 attributes were determined and entered into the MADM phase.

Iran ranks the highest in the Middle East in terms of mobile use. In Iran, 8 million people have a smartphone, and 83 million official mobile subscriptions have been registered, which is more than the country’s population. The growing prevalence of non-communicable diseases, such as diabetes, cardiovascular diseases, and their complications, requires an affordable tracking system available to everyone ^14^.

Mobile-health had advantages such as increased access to health care services, increased satisfaction of clients, and reduced cost of receiving services. Hence it can play an active role in the promotion and improvement of health in society. On the other hand, challenges facing the use and implementation of Telemedicine, such as security and confidentiality of the information and the way of paying health care costs, necessitate a national plan and program to overcome these challenges and successfully achieve the set goals ^15^. If these tools are utilized in Iranian medical centers, one can hope that, in addition to reduced medical errors and the costs for society members, the treatment stages can be carried out faster with better quality and efficiency ^1^.

A Health Technology Report showed that all analyses mainly related to economic evaluations predicted high savings from implementation Electronic Health Records in the health systems ^16^. This result can be compared with our results which “apps related to the access of physicians to health information of patients” had highest priority.

The results of a study on investigating the applications of mobile health and their communication infrastructure indicated that the criteria impacting the mobile health technological communication infrastructure are categorized in three main categories of "level of data transfer in mobile communication network", "level of application services in mobile communication network” and “level of access to mobile communication network”. Moreover, in the ranking of applications, “level of access to mobile communication network “is ranked first with the highest weight, and "level of data transfer in a mobile communication network" and “level of access to mobile communication network” are ranked second and third, respectively. The authors of this study prioritized mobile health apps in Iran by using the Fuzzy-AHP method. The most significant applications were for mobilization of society, electronic health records, and patient monitoring ^17^. Two cases of electronic health record and patient monitoring have common concepts with first two priorities of the current study, apps related to the access of physicians to health information of patients and apps related to the recording and tracking of vital signs, which indicates their high priority in the Iranian health system.

According to the results of this study, first three priorities of the Iranian health system for development of application in this field are 1) Apps related to the access of physicians to health information of patients, 2) Apps related to the recording and tracking of vital signs, and 3) Apps related to the support of electronic decision-making in health-related issues.

Apps related to the access of physicians to health information of patients can be defined as apps responsible for storing the information about each person's health and quickly providing the physician with this information through the application if needed. Mobile health allows experts to access patient data and various resources that significantly contribute to diagnosis and treatment ^18^.

In apps related to the recording and tracking of vital signs, the recorded data of significant events can form the basis for the statistics system of vital events, which includes an integral part of the health information system of countries. Health apps are developing and expanding to help manage chronic diseases and provide initial counseling on the disease. While smartphone technology improves the safety and outcome of the patient, at the same time, it also leads to a higher level of productivity ^19^. Individuals can use m-health to access health resources, and patients can use it for self-control and send care providers information such as blood pressure, data such as blood sugar for controlling diabetic patients or send a photo of a wound ^18^.

Decision-making support systems are information technology applications that can assist therapists in making correct and timely decisions about patients ^1^.

Considering the need for a proper decision at the right time, the presence of a system helping people in decision-making can be of great value. The information systems do not just provide information; they also participate in simple activities such as decision-making in any organization, in which they are known as decision-making support systems ^20^.

Clinical (or medical) decision-making support systems are interactive computer programs designed to help physicians and other health experts in the task of decision-making. The physician can interact with this system and get help from it in analyzing the patient’s data, diagnosis, prescribing, and other clinical activities ^20^. Apps related to the support of automated decision-making in health-related issues are defined in this area.

## Conclusion

The most important priorities for expanding the mobile application in the Iranian health field are: Apps related to the access of physicians to health information of patients, Apps related to the recording and tracking of vital signs, Apps related to the support of electronic decision-making in health-related issues, Apps related to the provision of health cares for remote areas, Apps related to the improvement of the service quality, Apps related to the selection of appropriate health behavioral patterns, Apps related to the improvement of society awareness and behavior on health issues, and Apps related to the increased knowledge of patients about disease.

By providing the necessary infrastructures for the Internet and national information networks, the health system can maintain and improve society’s health and also prevent inappropriate and unnecessary costs by designing and investing in the development of apps with priority. Organizations and centers responsible for health care in the society can provide and support the basis for the emergence of active startups in this area by adopting a futuristic and strategic approach.

There is an increasing demand for the use of health apps across the world, which itself emphasizes the use of a multi attributes approach for decision-making on choosing the appropriate technology in the field of mobile health in the related areas.

## Conflict of interest

The authors declare that there is no conflict of interest.

## Funding

This research was supported financially by Tehran University of Medical Sciences, School of Public Health.

## Highlights

- The most significant weight for priority setting attributes was related to “the feasibility of monitoring activities”.
- The least significant weight for priority setting attributes “the feasibility of access to apps in any location”.
- The apps related to “the physicians' access to patients’ health information” had the highest priority.
- The apps related to “the increased knowledge of patients about the disease” had the least priority
